# Sensory Specific Desires. The Role of Sensory Taste Exposure in Desire for Food with a Similar or Different Taste Profile

**DOI:** 10.3390/foods10123005

**Published:** 2021-12-04

**Authors:** Nora Chaaban, Barbara Vad Andersen

**Affiliations:** Food Quality Perception and Society, Department of Food Science, Aarhus University, Agro Food Park 48, DK 8200 Aarhus, Denmark; ncho_on@hotmail.com

**Keywords:** sensory specific desires, sensory taste profiles, food choice, sweetness, saltiness, appetite

## Abstract

The present study investigated how the sensory taste profile of a meal altered the subjective desire, wanting and liking of foods with a sweet, salty, sour, bitter, fatty, and spicy sensory profile, respectively. Participants (*n* = 85) ate a meal with a pronounced sensory taste profile: (1) sweet, (2) salty, or (3) sweet and salty combined. Self-reports of appetite, pleasantness, and sensory specific desires (SSD) were evaluated over the course of the meal using VAS-scales. SSDs were further studied through alterations in liking and desire for food samples with the main sensory profile being sweet (peach), salty (pretzel), sour (green apple), bitter (dark chocolate), fatty (whipped cream), and spicy (chilli nut), respectively. Consumption of food with a pronounced sensory taste profile was found to suppress the desire for food with a similar sensory taste profile, while the desire for different sensory profiles were enhanced or not affected. Further, when exposed to two pronounced tastes within the same meal, suppression of sensory desires was not only specific for the exposure tastes but tended to go beyond the sensory exposure. The findings suggest that taste variation within a meal holds the potential to create more satisfying meals, which can hinder additional desires after a meal and thus, lower additional calorie intake.

## 1. Introduction

Human eating behaviour is a broad term used to describe the overall processes of eating and drinking. Every day humans consider the simple questions about “what to eat?” and “how much to eat?” However, answering these questions is not as simple. Food intake and food choices are based on many underlying decision-making mechanisms [1,2,3], which is reflected in the increase in number of publications addressing how multiple internal and external factors influence human eating behaviour. Among these factors are personal, social, and cultural factors, as well as physiological, biological, and psychological, which all contribute to the complexity of the human eating behaviour [1,2,3].

Within the last decades, researchers have been exploring the link between sensory properties of food, i.e., taste, flavour, smell, texture, appearance, and human eating behaviour, revealing interesting aspects of appetite regulation [4,5,6]. Before a meal, the sight and smell of food can induce appetite. In particular, exposure to food odours, such as the smell of pizza or warm cookies, can stimulate salivation [7,8], induce appetite and increase food intake [9,10]. This suggests that odours can direct appetite and food choices towards foods that are signalled by the odour specifically [11], a concept known as sensory specific appetite [10]. During a meal, a satiation can develop that is specific to the food eaten. This concept is known as sensory specific satiety, and is observed as a decrease in hedonic evaluation for a food eaten as compared to a food not eaten [12]. Sensory specific satiety, is driven by the sensory properties of food, for example taste, texture, colour, shape and temperature [13]. Transfer effects have been observed, where eating a food with a pronounced taste profile to satiation e.g., sweet or salty [14,15], reduced hedonic evaluation of foods sharing the pronounced taste profile. However, not only sensations that demotivate eating develop during a meal; feelings of ‘wanting’ for a certain sensory exposure can develop during a meal. These sensations are known as sensory specific desires (SSDs), and can be described as the intrinsic motivation for consuming foods with a specific sensory profile [16,17], e.g., sweet, salty, sour, bitter, fatty or spicy. Therefore, SSDs include and go beyond specific foods. Recent studies [16,17,18] have indicated an association between the sensory taste profile of an eaten food and future food choices through the development of SSDs. SSDs are believed to motivate food seeking behaviours [19] in the search for satisfaction, and can lead to consumption of additional calories after a meal [16]. The effect of developed SSD on liking and wanting of foods with similar and different sensory profiles, respectively, has been demonstrated in studies on spicy soup [16] and sweet yoghurt [17], and more implicit in studies on sweet and savoury food [20] and in sweet drinks [21]. Overall, these studies showed a decrease in pleasantness and wanting of foods that share a similar taste sensory profile with an eaten food, and either an enhanced or unaffected desire for foods with a different sensory taste profile. The effect of SSDs as they develop during a meal on actual food choices has to the authors’ knowledge only been demonstrated in one study by Duerlund et al. (2021). In this study, participants with an initial desire for a salty late-afternoon snack showed a clear tendency of choosing a salty snack over a sweet snack, and vice versa [18]. Thus, the topic of SSDs in shaping postmeal eating behaviour need to be explored further to strengthen the scientific evidence. Further, more research on the topic is needed to clarify if this knowledge can be used to design meals that maximise sensory satisfaction and reduce intake of postmeal additional calories.

The overall objective of this study was to elucidate how food with pronounced sensory taste profiles generate SSDs and affect hedonic evaluation of foods with similar and different sensory profiles. Specifically, the study aimed to investigate how the sensory taste profile of a sweet and salty meal, respectively altered the subjective desire for wanting and liking of foods with a sweet, salty, sour, bitter, fatty, and spicy sensory profile. Secondly, this study aimed to investigate the effect of increasing sensory complexity i.e., a combined sweet and salty meal compared to a pronounced sweet or salt meal, respectively, affected the development of desire for, wanting and liking of foods with a sweet, salty, sour, bitter, fatty and spicy sensory profile.

## 2. Materials and Methods

### 2.1. Participants and Recruitment

The Central Denmark Region Committees on Health Research Ethics approved the study. The study was carried out at Aarhus University, Denmark and Agro Food Park, Denmark on a total of 89 healthy adult participants. Participants were recruited trough the posting of recruitment flyers at several locations around Aarhus University campus and library, along with recruitment posts on various social media including LinkedIn and Facebook. Further, participants were recruited through direct face-to-face contact at the library and around campus. Inclusion criteria were; Danish- or English-speakers, non-food allergy sufferers, and willing to taste different foods while answering a questionnaire. Due to missing data (discovered during data analysis), four participants were excluded from this study. Thus, the study relies on data from a total of 85 participants. Characteristics of the participants are summarized in Table 1 below.

### 2.2. Test Stimulus

The study aimed to investigate alterations in SSD as caused by the consumption of a: (1) pronounced sweet meal (banana), (2) pronounced salty meal (salty crackers), or (3) pronounced sweet and salty meal combined (banana + salty crackers) to satiation. The following sections provide a description of each meal.

#### 2.2.1. Sweet Meal Exposure

Organic bananas (Fairfresh, Dominican Republic, type: Cavendish, class 1; per 100 g: 356 KJ (85 kcal), 1.2 g protein, 0.3 g fat, 20 g carbohydrate) were bought 1–2 days prior to the experiment day to ensure ripeness for more sweetness. Only appealing bananas with no brown spots were used. During the experiment, bananas were cut in halves and each half was served in transparent beakers. One serving consisted of a half banana (average weight = 114 g). In total 10 servings were offered to the subjects during the sweet meal exposure.

#### 2.2.2. Salty Meal Exposure

Multiple packs of salty crackers (‘TUC Crackers’, Mondeléz International, Chicago, IL, USA. Per 100 g; 1940 KJ (465 kcal), 8.10 g protein, 19 g fat, 65 g carbohydrate) were bought and stored at room temperature. During the experiment, each serving consisted of two crackers (average weight = 7 g), which were served in a transparent beaker. In total 10 servings were offered to the subjects during the salty meal exposure. 

#### 2.2.3. Sweet + Salty Meal Exposure

The sweet + salty exposure consisted of bananas and crackers as applied in the sweet and salty meal, respectively (Section 2.2.1 and Section 2.2.2). One serving (average weight = 61 g), consisted of one quarter of a banana (average weight = 57 g) along with one cracker (average weight = 4 g) which were prepared during the experiment and served in a transparent beaker. As with the sweet meal, bananas were bought 1–2 days prior to experiment, and only appealing bananas with no brown spots were used. In total, 15 were given to the subjects during the sweet + salty meal.

### 2.3. Food Samples with Specific Sensory Profile

Six food samples: (1) peach, (2) green apple, (3) pretzel, (4) dark chocolate, (5) spicy nut, and (6) whipped cream, were chosen from a previous studies [16,17], where an experienced sensory panel documented the pronounced sensory profile of several foods. Each food sample represented one specific sensory profile; (1) sweet, (2) salty, (3) sour, (4) bitter, (5) fatty and (6) spicy, respectively (Table 2). Food samples were served before and after the meal exposures (sweet, salty, or sweet + salty combined) to study if potential alterations in SSDs tracked onto evaluation of foods with pronounced sensory taste profiles. 

### 2.4. Procedure

The experiment ran over a period of two weeks. Consent was obtained from all participants. During recruitment, participants were randomly distributed to the sweet, salty, and sweet + salty exposure, respectively, ensuring a balanced number of participants in each group. All participants were instructed to fast for two hours prior to experiment session. The experiment consisted of six parts, as outlined below, where different questionnaires were presented and answered using a 15 cm visual analogue scale (VAS). In the experiment room, walls were placed between participants to avoid interaction and disturbance between participants. The experiment lasted approximately one hour in total. The Central Denmark Region Committees on Health Research Ethics approved the study being conducted.

(1) Introduction: Upon arrival, participants were introduced to the experiment by receiving an overview of the experiment steps along with the questionnaires. Further, participants were instructed in how to use a VAS scale. The purpose of the experiment was not explained.
(2) Serving and rating of food samples with a pronounced sensory profile: Each participant was served six food samples (peach, pretzel, green apple, dark chocolate, whipped cream and chilli nuts, Table 2), and instructed to taste the food samples in the order presented in questionnaire. The order was randomized between participants, however, the food samples were presented in the same order for each participants both before and after the meal exposure (sweet, salty or sweet + salty). Immediately after eating a food sample, participants were instructed to rate their liking of the food sample (“How much do you like the sample right now?”) and their wanting for more (“If you were offered another portion, how much would you then want to eat it?”). One cup of water was provided for palate cleansing before proceeding to the next step.
(3) Rating of appetite sensations: At baseline, participants were instructed to rate their overall feeling of hunger (“How hungry are you right now?”), satiation (“How satiated are you right now?”), general liking of the food presented in main meal (banana, crackers or both) (“How much do generally like banana/crackers?”), and their desire for the main meal (“How much do you want to eat banana/crackers right now?”).
(4) Serving and rating of the meal with a pronounced sensory profile: Depending on the exposure, participants were served either banana (sweet meal), salty crackers (salty meal) or banana + salty crackers combined (sweet + salty meal). Beginning at the same time, participants were served one serving of the meal, and were instructed to eat the whole serving before making their rating. Afterwards, participants were instructed to evaluate liking (“How much do you like the banana/crackers right now?”), sensory profile perception (“What is the dominating taste?” (Sour, sweet, salty, bitter, fatty, spicy)), hunger, satiation and desire for more of the meal. Further, participants were asked to rate their desire for eating something sweet, salty, sour, bitter, fatty, and spicy (“How much do you want to eat something sweet/salty/sour/bitter/fatty/spicy?”). This step was repeated until the participant was comfortably satiated (limit was set at maximum 10 servings of banana or salty crackers, and 15 servings of banana + salty crackers). This allowed the study of dynamics in palatability, appetite and desires as they developed over the meal. When reaching the state of comfortably satiated, participants were not served further servings of meal, and proceeded to next step.
(5) Evaluation of subjective sensory desires: Upon reaching the state of “comfortable satiation”, participants were served the food samples once again in the same order as in step 2, and where were instructed to evaluate liking and wanting for food samples.
(6) Demographics: At the end of the test day, participants filled out a final questionnaire regarding; weight, height, age, gender, and educational level. They were thanked for their participation and assured anonymity. To minimize disturbance, participants were asked to stay in the room until all participants had completed the experiment.

### 2.5. Statistical Analyses 

Two-way ANOVA was used to analyse significant differences in SSDs (sweet, salty, sour, bitter, spicy, and fatty), and liking and desire of the corresponding food samples (peach, pretzel, apple, dark chocolate, chilli nut and whipped cream, respectively). The ANOVA model included the two factors: time (baseline vs. postmeal) and meal (sweet, salty, and sweet + salty combined), as well as their interaction. As the experiment was performed on three different groups of participants (one group for each meal), ANOVA and post hoc analysis were conducted as nonrepeated measures and unpaired *t*-tests. Further, paired *t*-tests analysis was used to determine significant difference in various response variables, including appetite sensations, liking and desire, within each group, i.e., meal, over time (baseline vs. postmeal).

Paired *t*-test analyses was carried out in Excel (Microsoft, 16.35, Washington, WS, USA), and ANOVA and post-hoc analyses was carried out in XLstat (Addison, January 2020, New York, NY, USA). The significance level was set at p≤0.05. Descriptive statistics: mean values and standard deviations, were used to analyse participant characteristics such as gender, age, BMI and educational level.

## 3. Results

### 3.1. Sensory Perception of the Exposure Meal

To study if the taste of the exposure meals (banana, salty crackers and banana + salty crackers, respectively) was perceived as expected (sweet, salty and sweet + salty, respectively), participants were asked to indicate their primary taste perception of the received meal. The majority of the participants (71%), who received the sweet meal, perceived banana as primarily sweet. Fewer perceived the banana as primarily fatty (25%) or primarily bitter (4%). For the salty meal, the majority of the participants (77%) perceived crackers as primarily salty, and a few perceived the crackers as primarily sweet (8%), spicy (8%) or fatty (8%). These results indicate that the chosen foods were perceived as expected for the majority of the participants. 

### 3.2. Effect on Appetite, Liking and Desire Ratings

Prior to intake of the exposure meal, no significant difference was found in initial hunger and satiety ratings between three conditions; banana, salty crackers and banana + salty crackers combined. On 15 cm VAS scales anchored from “not at all” (0 cm) to “extremely” (15 cm), the initial mean ratings of hunger were 8.8 cm (±3.4 cm), 9.6 cm (±3.4 cm) and 9.6 cm (±3.2 cm) for banana, salty crackers and banana + salty crackers exposure groups, respectively. This indicates that participants in all three conditions were moderately hungry before consumption of the exposure meal.

**Figure 1:**
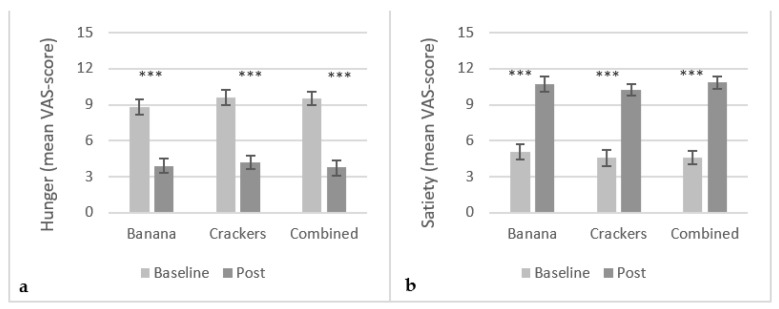
Mean VAS-score (15 cm scale) on subjective hunger (**a**) and satiety (**b**) ratings in the conditions; banana (N = 29), crackers (N = 28) and banana+crackers combined (N = 28). Error bars are in Standard Error of Mean (SEM). ***: p<0.0001.

As a consequence of exposure meal intake, ratings of hunger significantly decreased (p<0.0001) in all three conditions (Figure 1a) while ratings of satiety significantly increased (p<0.0001) in all three conditions (Figure 1b). No significant difference in hunger and satiety ratings was found between the three conditions post intake.

**Figure 2:**
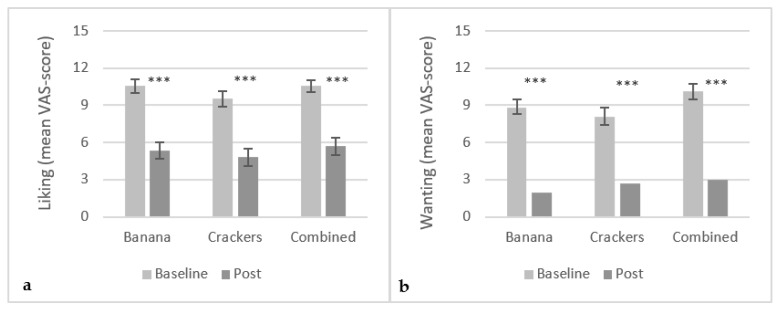
Mean VAS-score (15 cm scale) on subjective liking (**a**) and desire (**b**) ratings in the conditions; banana (N = 29), crackers (N = 28) and banana+crackers combined (N = 28). Error bars are in Standard Error of Mean (SEM). ***: p<0.0001.

Initial mean liking ratings of banana, salty crackers and banana + salty crackers were 10.6 cm (±3.0 cm), 9.6 cm (±3.2) and 10.5 cm (±2.4 cm), respectively, indicating that the chosen foods were well-liked by the participants. No significant difference was found in the initial liking ratings between the three conditions. After intake of the exposure meal, subjective rating of liking significantly decreased in all three groups (p<0.0001) (Figure 2a). Similarly, the desire for another portion of the exposure meal significantly decreased with continued intake (p<0.0001) (Figure 2b). Postintake, no significant difference was found in liking and wanting ratings between the three conditions. 

### 3.3. Effect on Sensory Specific Desires

Prior to intake of the exposure meal (banana, salty crackers, or banana + salty crackers combined), no significant difference was found in the liking and desire for the food samples; peach, pretzel, apple, dark chocolate, chilli nut and whipped cream. However, postmeal intake differences in desire for the taste profiles (sweet, salty, sour, bitter, fatty and spicy), and liking and desire for food samples were found, as elaborated below.

#### 3.3.1. Effect of the Sweet Exposure Meal

**Figure 3:**
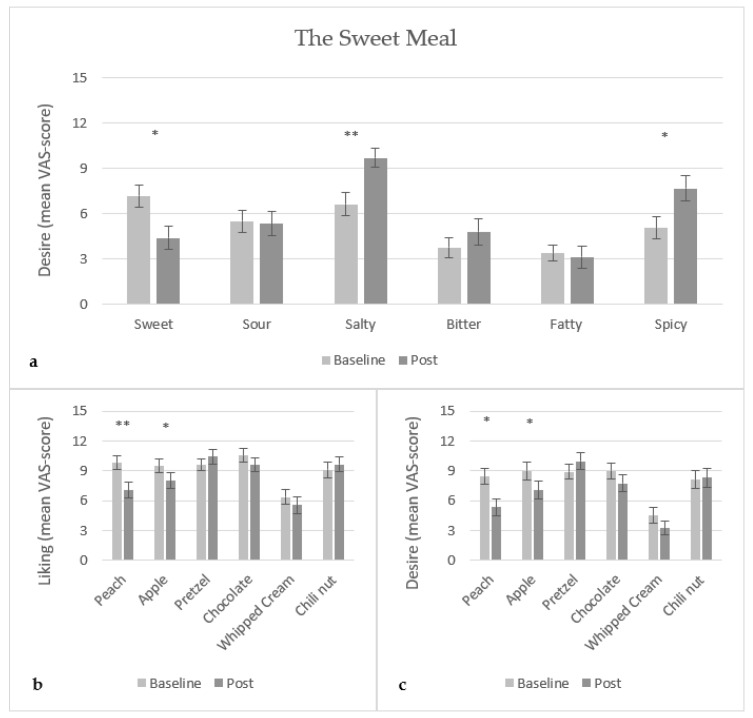
Mean VAS-score (15 cm scale) of (**a**) desire of food with specific taste, (**b**) liking of food samples, and (**c**) desire of food samples, for the sweet meal (banana) (N = 29). *: 0.01<p<0.05, **: 0.0001<p<0.001.

Intake of the sweet meal (banana) led to a significant decrease (p<0.01) in the desire for sweet food (Figure 3a). This finding was reflected in a decreased liking (p<0.001) (Figure 3b) and decreased desire (p<0.01) (Figure 3c) of the sweet food sample peach. Further, a significant increase in the desire for salty (p<0.001) and spicy (p<0.01) food was found, which was however not reflected in either of the corresponding food samples (pretzel and chili nut, respectively). A decrease in the liking (p<0.05) and desire (p<0.01) of the green apple food sample was found. However, this finding was not reflected in the corresponding desire of sour food. For the bitter and fatty taste categories and their corresponding food samples, dark chocolate and whipped cream, respectively, no significant difference was found in liking and desire ratings.

#### 3.3.2. Effect of the Salty Exposure Meal

**Figure 4:**
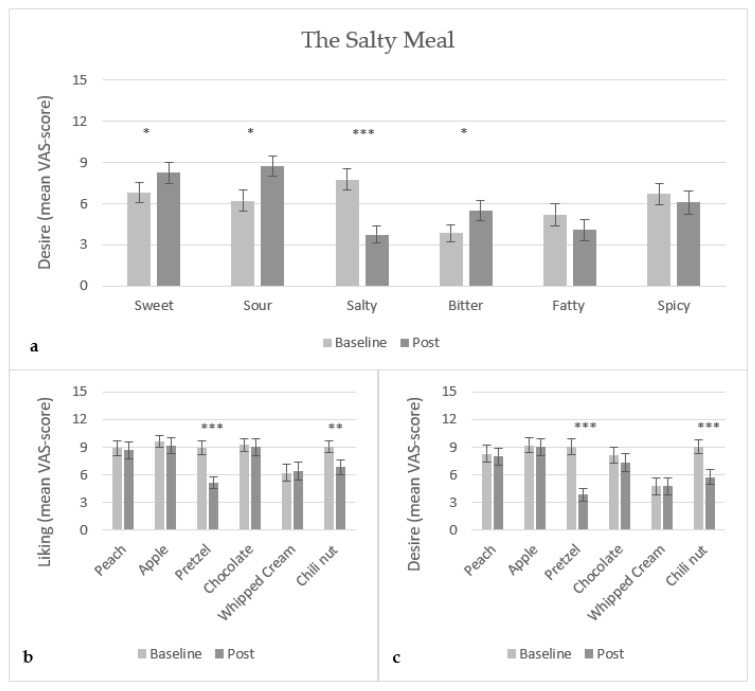
Mean VAS-score (15 cm scale) of (**a**) desire of food with specific taste, (**b**) liking of food samples, and (**c**) desire of food samples, for the salty meal (crackers) (N = 28). *: 0.01<p<0.05, **: 0.0001<p<0.001, and ***: p<0.0001.

Consumption of the salty meal (salty crackers) led to a significant decrease (p<0.0001) in the desire for salty food, which was reflected in the decreased liking (p<0.0001) and desire (p<0.0001) of the corresponding salty food sample, pretzel. Further, a significant increase in the desire for sweet food (p<0.05), sour food (p<0.01) and bitter food (p<0.05) was found (Figure 4a), which was, however, not reflected in the liking (Figure 4b) or desire (Figure 4c) for the corresponding food samples; peaches, green apple and dark chocolate, respectively. A significant decrease in the liking (p<0.001) and desire (p<0.0001) for chilli nut was found, but there was no significant difference in the desire for spicy food. No significant difference was found in the desire for fatty food and liking and desire for the corresponding fatty food sample, whipped cream.

#### 3.3.3. Effect of the Combined Sweet and Salty Exposure Meal

**Figure 5:**
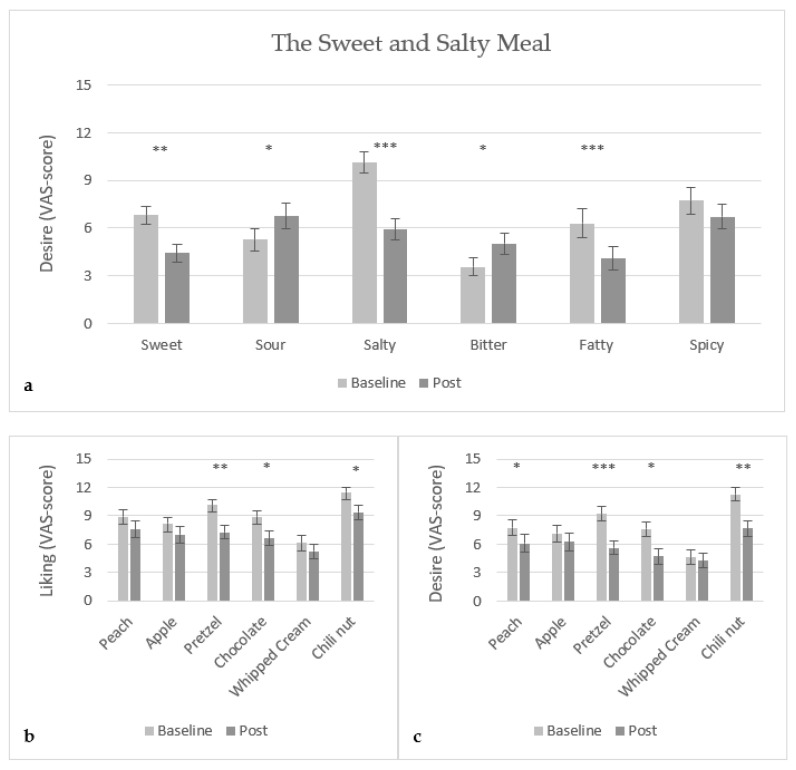
Mean VAS-score (15 cm scale) of (**a**) desire of food with specific taste, (**b**) liking of food samples, and (**c**) desire of food samples, for the sweet and salty meal (banana + crackers combined) (N = 28). *: 0.01<p<0.05, **: 0.0001<p<0.001, and ***: p<0.0001.

Intake of the combined sweet and salty meal led to a decrease in desire for both sweet food (p<0.001) and salty food (p<0.0001) (Figure 5a). For the corresponding sweet and salty food samples, peach and pretzel, the desire ratings were also significantly lower (peach, p<0.05 and pretzel, p<0.0001) (Figure 5c). For the liking ratings, only a significant decrease was found for the salty pretzel (p<0.001) (Figure 5b). A significant increase (p<0.05) in the desire for sour food was found, which was not reflected in the liking nor desire of sour green apple. Further, consumption of the sweet and salty combined meal led to an increase in the desire of bitter food (p<0.05). However, this finding was contrary to the findings in liking and desire found for the corresponding bitter food sample, dark chocolate, as a significant decrease in both liking (p<0.01) and desire (p<0.01) was found. The desire for fatty food significantly decreased (p<0.001), but no significant difference was found for the corresponding fatty food sample, whipped cream. Lastly, the liking (p<0.01) and desire (p<0.001) for the spicy chili nut significantly decreased, while there was no significant difference in the desire for spicy food.

### 3.4. Comparison of Sensory Desires between the Exposure Meals

By comparing the postmeal desire ratings for the taste categories (sweet, salty, sour, bitter, fatty, and spicy), differences were found between the three conditions (sweet, salty, sweet + salty combined) (Table 3). A significant higher desire for something sweet (p<0.01) and sour (p<0.05), and a significant lower desire for something salty (p<0.0001) was found after exposure to a salty meal compared to the sweet meal. Only for the corresponding salty food sample, pretzel, a significant lower rating in liking (p<0.0001) and desire (p<0.0001) was found after exposure to the salty meal compared to the sweet exposure meal. Further, a significant lower desire for something salty (p<0.01) was found after exposure of sweet + salty combined meal compared to the sweet exposure meal, which was likewise reflected in the lower ratings of liking (p<0.01) and desire (p<0.001) for pretzel. A significant lower rating in the liking (p<0.05) of dark chocolate was found after exposure to the sweet + salty meal compared to the sweet exposure meal. Comparing the salty and sweet + salty meal, only a significant lower desire for something sweet (p<0.01) was found after exposure to the sweet + salty meal. No main effects were found in the remaining response variables.

## 4. Discussion

### 4.1. Effect on Sensory Specific Desires

In this study, we investigated how sensory desires develop during a meal based on the primary sensory taste experience, and if the desires transfer into desire and liking for food samples showing the desired sensory characteristic. Evaluating the development of SSDs during consumption of a meal is relevant, as these desires can influence postmeal food choice and intake [18], despite feeling full, and thus lead to a positive energy balance in the longer term [22].

Of key relevance, this study showed that the sensory profile of an eaten food affects the subjective desire for foods sharing the sensory taste profile and differing in sensory taste profile. These findings suggest that sensory perceptions lead to suppression and development of SSDs during a meal. More specifically, consumption of food with a pronounced sensory taste profile suppressed the desire for food with a similar sensory taste profile, while the desires for different sensory profiles were either increased or not affected. In the present study, it was found that the consumption of a pronounced sweet meal decreased the subjective desire for other sweet foods. Similarly, the consumption of a pronounced salty meal decreased the desire for other salty foods. Supporting this view, Rolls and Hetherington (1989) have previously described that transfer effects can be observed to uneaten foods that are similar, e.g., share same sensory profile, to an eaten food [23]. Likewise, this phenomenon has been observed in previous studies, for instance, the consumption of a spicy soup led to a decrease in the desire for spicy foods [16], and consumption of a sweet yoghurt led to a decrease in the desire for sweet foods [17]. In present study, the decreased desire for sweet and salty, respectively, was reflected in the liking and wanting for food samples sharing the sensory taste profile (sweet peach and salty pretzel, respectively). These findings indicate that the desire is not only on a mental level but transferred into perceived pleasantness and wanting for actual foods, leaving the hypothesis that these foods are less likely to be chosen afterwards. Humans are omnivores meaning that we have the ability to eat a variety of food, but at the same time we cannot survive on one food source only. Hence, we need to eat a variety of food to obtain various essential nutrients. From an evolutionary perspective, the decreased desire for food sharing the same sensory characteristics as a food just eaten, can serve as a mechanism to ensure variation in nutrient intake. It has been hypothesized that the sensory taste profiles associate with the presence of nutrients in the food, and perception of the sensory cues thus serves to adjust intake [6]. This reasoning can explain the decreased desire for foods sharing the same pronounced sensory taste profile as food just eaten, which was found in the present study, and further, why our participants despite feeling full, still developed desires for food with a different sensory profile. Previous research has found similar results. Duerlund et al. (2019) [24] found that consumers experienced fullness and sensory desires simultaneously. In later studies, they found that participants, though feeling full, still experienced a desire for something sweet after consuming a nonsweet meal [25], and that an initial desire for something sweet or salty resulted in choosing snacks with the desired sensory profile, a sweet snack or a salty snack, respectively. The negative association between sensory taste exposure and later choice of food with a similar sensory characteristic leaves the question of whether sensory complexity enhancements within a meal (e.g., by increasing the number of pronounced sensory taste characteristics) can serve to limit desire for additional intake, and thus be used as a strategy to decrease additional intake (e.g., intermeal snacking behaviour). This will be discussed further in Section 4.3.

Furthermore, the present study showed that sweet and salty desire was mutually driven by sweet and salty exposure during a meal, as consuming a sweet meal led to an increase in the desire for salty foods, and vice versa. From a health perspective, these findings can be concerning due to an overall high intake of sugar and salt in the Western diet [26]. It can be speculated that high sugar intake drives high salt intake and vice versa, leading to a continued unhealthy eating pattern. However, considering the results from the food samples in the present study, neither of the increased desires were reflected in liking and wanting for the corresponding food samples (sweet peach and salty pretzel, respectively). 

Besides driving a desire for salty food, exposure to a sweet meal was found to increase the desire for spicy foods. The link between sweetness and spiciness has been explored previously in a study by Andersen et al. (2017), where it was found that spicy food increased the desire for sweet foods, indicating a mutual link between exposure and desire for sweetness and spiciness, similar to what has been found for saltiness and sweetness. Exposure to sweetness, further led to decreased liking and wanting for sour apple. This finding supports the result of a study by Olsen et al. (2011), where consumption of sweet yoghurt likewise lead to a decreased desire for sour food. 

In the present study, consumption of a salty meal led to an increase in the desire for something sour (and sweet as discussed above), and a decrease in desire for something bitter. To the authors’ knowledge, the effects of a salty meal on SSDs have not been explored previously, and thereby the study brings new knowledge, which deserves further investigation. 

### 4.2. Comparison of SSD between Simple Food vs. Complex Food

The present study further investigated the effect of sensory complexity enhancement, by exposing participants to foods with a pronounced salt and sweet taste combined, on SSDs, and desire and liking for food samples with similar and different sensory taste profile. Key findings showed that a combined sweet and salty meal, led to a decrease in desire for both sweet food and salty food, and the results could be retrieved in liking and desire ratings for food samples sharing these sensory characteristics (though not significant for liking of sweet peach). This supports the view that exposure to certain sensory properties in one meal, decrease the subjective drive to engage in following consumption of foods sharing these sensory properties. Similar results have been found in a study by Olsen et al., where yoghurt, which was perceived as sweet, sour, and fatty caused a decrease in desire for sweet, sour, and fatty foods [17].

When exposed to both a combined, dominating sweet and salty taste, the desire for fatty food also decreased. These results can, at least partly be explained by the meal being perceived as fatty by 7% of the participants. Thereby, transfer effects from the perception of the ‘fatty’ stimuli could result in a lower desire for fatty foods. Interestingly, liking and desire ratings for all foods samples likewise decreased (i.e., samples holding a pronounced sweet, salty, sour, bitter, fatty and spicy taste profile, respectively). Note, whether the effect was significant depended on the food sample in focus. These findings indicate that sensory complexity negatively correlates with additional food desires, a process that can be hypothesized to involve a faster fullness response. An increase in satiation and lower hunger levels were indeed found after exposure to a more complex meal, but from the present study it is not clear if the more sensory complex meal resulted in a faster satiation than more sensory simple meal. In a study by Andersen and colleagues (2017) it was found that the addition of cayenne pepper, increasing sensory complexity via oral heat, resulted in higher satiation at the end of the meal and one hour later [16], but more research is needed to clarify if and how sensory complexity drives satiation. It can be hypothesized that increases in sensory complexity leaves consumers with a higher feeling of satisfaction and thus not feeling an urge to continue intake. A higher sensory satisfaction after intake of the more sensory complex meal, was likewise reported in the study by Andersen and colleagues [16]. The authors explain that sensory satisfaction can be seen as a state of contentment where sensory desires are fulfilled [27,28], which logically can be associated with a low need and desire to continue eating. This area of research deserves further exploration, e.g., future studies are needed to clarify, if simply increasing the complexity or the intensity of sensory taste stimuli in a meal can bring sensory satisfaction and lower post meal intakes, or if the effects function around an optimum depending on subjective preferences. 

Another interesting finding is, when combining the sweet and the salty tastes in a meal, the salty taste appear to be more dominant on the alterations of SSD than the sweet taste. This finding was observed when comparing the three conditions, where desires after the combined meal were more in line with the desires after the salty meal than after the sweet meal: an increase in the desire for something sour and something bitter and a decrease in desire for something salty and the food samples pretzel and chilli nut. These results are in alignment with a previous study by Griffioen-Roose (2010), who found that savoury foods have a stronger modulating effect on appetite compared to sweet food.

### 4.3. Limitations

Research in the area of SSD can provide a greater understanding of the drivers involved in human food choice behaviour. This knowledge can be beneficial for future design of more satisfying and less energy-dense meals in the aim of improving public health and controlling obesity. From the findings in present study, it is evident that the sensory taste profile of eaten foods alters SSDs, and more interestingly, that the combination of tastes presented in a meal hold a suppressive effect on SSDs. However, the present study did not include a postmeal free choice of a snack. Therefore, we cannot conclude whether the SSDs actually affect snacking choice and intake. To be able to measure this, future studies could include a setup where participants were offered various snack choices after intake of the meal. Each snack option could hold a pronounced sensory profile, to clarify if SSDs were reflected in snack choice behaviour. Another limitation in this study relates to the higher number of female participants compared to male participants. To be able to generalize the results over both genders, future studies should strive for inclusion of male participants. 

Overall, in this study, alterations in sensory specific desires were not necessarily reflected in the corresponding food samples, and vice versa. One explanation is that SSD can be stimuli specific, e.g., depend on the exact type of sweetener. This aspect was addressed in a previous study by Andersen et al. (2017), where participants showed a significantly lower desire for the chilli nut food sample but not wasabi beans after consumption of a spicy soup, although both food samples represented the spicy sensory profile. Furthermore, whole foods were used as representatives for foods with a pronounced sensory profile. Specifically, bananas were used as a sweet meal stimuli, and salty crackers were the salty meal stimuli. Though the foods indeed do hold a pronounced sweet and salt sensory taste profile, respectively, both foods do also hold a variety of other sensory properties including different aroma and texture-related properties. It cannot be ruled out that these properties influenced the results. Further, as participants in this study were naïve consumers, who are not necessarily used to applying sensory terms in their daily vocabulary, it cannot be ruled out the sensory attributes e.g., sweet and salty, and the following results on sensory characteristics and SSDs are biased from participants’ own understanding of the sensory terms.

## 5. Conclusions

The present study focused on alterations in SSDs after consumption of a meal with a pronounced sensory taste profile (either sweet, salty, or sweet and salty combined). In conclusion, the findings provide confirmatory evidence that there is an interaction between exposure to sensory taste characteristics and suppression and development of SSDs. Consumption of food with a pronounced sensory taste profile suppressed the desire for food with a similar sensory taste profile, while the desire for different sensory profiles was enhanced or not affected. These results point to the fact that the sensory experience of eating is an important factor to understand the complexity of human eating behaviour. 

Key results revealed that consumption of a sweet meal caused a desire for salty foods, and vice versa. Further, the results showed that desires were transferred into liking and desire for actual foods with a similar sensory taste profile as the consumed food. For instance, consuming a sweet meal led to a decrease in the desire for other sweet foods, such as peach, which confirms theories about SSS.

Another key result revealed that exposure to two pronounced tastes within the same meal (sweet and salty) meant that suppression of sensory desires tended to go beyond the sensory exposures. These results deserve further exploration, as the findings suggest that taste variation within a meal holds the potential to create more satisfying meals, which hinders snacking behaviour and thus lower additional calorie intake, which is relevant in a world with alarming increases in obesity rates.

## Figures and Tables

**Table 1:** Summary of participant characteristics.

Characteristics	Sweet Meal Exposure	Salty Meal Exposure	Sweet + Salty Meal Exposure
Total number of participants (N)	29	28	28
Gender (Number of females/number of males)	23/6	23/5	25/3
Age (years) ^a^	28.5 (±19.8)	29.2 (±9.9)	31.2 (±12.1)
BMI (kg/m^2^) ^a^	23.3 (±4.7)	24.1 (±5.3)	22.6 (±4.6)
Educational level ^b^	4.4 (*n*_2_ = 6, *n*_4_ = 2, *n*_5_ = 13, *n*_6_ = 7, *n*_7_ = 1)	4.8 (*n*_2_ = 5, *n*_4_ = 1, *n*_5_ = 13, *n*_6_ = 9)	4.8 (*n*_1_ = 1, *n*_2_ = 2, *n*_4_ = 2, *n*_5_ = 12, *n*_6_ = 9, *n*_7_ = 1)
Overall liking of banana (cm) ^c^	10.1 (±2.4)	--	9.6 (±3.2)
Overall liking of crackers (cm) ^c^	--	9.0 (±3.4)	10.6 (±3.3)

^a^ Mean (std). ^b^ Number of participants (*n*) with completed level of education: (1) lower secondary, (2) higher secondary, (3) higher secondary with trainee, (4) short-length higher education, (5) medium-length higher education, (6) long higher education, and (7) other. ^c^ Mean (std) rating collected on a 15 cm visual analogue scale (VAS).

**Table 2:** Food samples used to represent the sensory profiles of sweet, sour, salt, bitter, spicy, and fat.

Sweet	Sour	Salt	Bitter	Spicy	Fat
Canned peach	Green apple with two drops of lemon juice	Pretzel	Dark chocolate	Chilli nut	Whipped cream

**Table 3:** Comparison of postintake ratings of desire for taste categories and liking and wanting for food samples between meals.

	Sweet Meal	Salty Meal	Sweet + Salty Meal
**Desire for:**			
Sweet	4.42 (±3.90) ^A^	8.23 (±4.02) ^B^	4.42 (±3.00) ^AC^
Salty	9.64 (±3.24) ^A^	3.72 (±3.24) ^B^	5.91 (±3.60) ^BC^
Sour	5.32 (±4.05) ^A^	8.72 (±3.88) ^B^	6.76 (±4.28) ^AB^
Bitter	4.86 (±4.63) ^A^	5.49 (±3.92) ^A^	5.04 (±3.52) ^A^
Fatty	2.97 (±3.8) ^A^	4.07 (±3.97) ^A^	4.10 (±3.97) ^A^
Spicy	7.48 (±4.41) ^A^	6.07 (±4.32) ^A^	6.71 (±4.14) ^A^
**Liking of:**			
Peach	7.02 (±4.27) ^A^	8.70 (±4.80) ^A^	7.57 (±4.41) ^A^
Pretzel	10.44 (±4.80) ^A^	5.41 (±3.42) ^B^	7.30 (±3.84) ^BC^
Apple, green	8.02 (±4.44) ^A^	9.11 (±4.52) ^A^	7.01 (±4.75) ^A^
Chocolate, dark	9.60 (±4.00) ^A^	9.03 (±4.83) ^AB^	6.60 (±4.03) ^BC^
Whipped cream	5.54 (±4.50) ^A^	6.41 (±4.93) ^A^	5.21 (±4.30) ^A^
Chilli nut	9.70 (±3.81) ^A^	6.81 (±4.52) ^A^	9.40 (±4.03) ^A^
**Desire for:**			
Peach	5.34 (±5.53) ^A^	8.00 (±4.90) ^A^	6.10 (±4.90) ^A^
Pretzel	10.00 (±4.50) ^A^	3.83 (±3.85) ^B^	5.61 (±3.90) ^BC^
Apple, green	7.04 (±4.80) ^A^	9.03 (±4.91) ^A^	6.30 (±5.05) ^A^
Chocolate, dark	7.49 (±4.61) ^A^	7.32 (±5.24) ^A^	4.71 (±4.41) ^A^
Whipped cream	3.21 (±3.71) ^A^	4.75 (±5.00) ^A^	4.30 (±4.12) ^A^
Chilli nut	8.31 (±5.00) ^A^	5.80 (±4.52) ^A^	7.70 (±4.3) ^A^

Mean VAS scores (std). Different letters within same row indicate significant difference.

## Data Availability

The data presented in this study is available on request from the corresponding author.

## References

[1] Lacaille L. (2013). Eating Behavior. Encyclopedia of Behavioral Medicine.

[2] Laviano A., Di Lazzaro L., Koverech A. (2018). Changes in eating behavior, taste and food preferences and the effects of gastrointestinal hormones. Clin. Nutr. Exp..

[3] Köster E. (2009). Diversity in the determinants of food choice: A psychological perspective. Food Qual. Prefer..

[4] Sørensen L.B., Møller P., Flint A., Martens M., Raben A. (2003). Effect of sensory perception of foods on appetite and food intake: A review of studies on humans. Int. J. Obes..

[5] Forde C.G. (2018). From perception to ingestion; the role of sensory properties in energy selection, eating behaviour and food intake. Food Qual. Prefer..

[6] McCrickerd K., Forde C. (2016). Sensory influences on food intake control: Moving beyond palatability. Obes. Rev..

[7] Engelen L., de Wijk R.A., Prinz J.F., van der Bilt A., Bosman F. (2003). The relation between saliva flow after different stimulations and the perception of flavor and texture attributes in custard dessert. Physiol. Behav..

[8] Ferriday D., Brunstrom J.M. (2011). ‘I just can’t help myself’: Effects of food-cue exposure in overweight and lean individuals. Int. J. Obes..

[9] Zoon H.F.A., De Graaf C., Boesveldt S. (2016). Food Odours Direct Specific Appetite. Foods.

[10] Ramaekers M.G., Boesveldt S., Lakemond C.M.M., Boekel M.A.J.S.V., Luning P.A. (2014). Odors: Appetizing or satiating? Development of appetite during odor exposure over time. Int. J. Obes..

[11] Proserpio C., de Graaf C., Laureati M., Pagliarini E., Boesveldt S. (2017). Impact of ambient odors on food intake, saliva production and appetite ratings. Physiol. Behav..

[12] Rolls B.J., Rolls E., Rowe E.A., Sweeney K. (1981). Sensory specific satiety in man. Physiol. Behav..

[13] Rolls B.J., Rowe E.A., Rolls E.T. (1982). How sensory properties of foods affect human feeding-behaviour. Physiol. Behav..

[14] Rolls B.J., Van Duijvenvoorde P., Rolls E. (1984). Pleasantness changes and food intake in a varied four-course meal. Appetite.

[15] Guinard J.X., Brun P. (1998). Sensory Specific Satiety. Comparison of taste and texture efefcts. Appetite.

[16] Andersen B., Byrne D., Bredie W., Møller P. (2017). Cayenne pepper in a meal: Effect of oral heat on feelings of appetite, sensory specific desires and well-being. Food Qual. Prefer..

[17] Olsen A., Ritz C., Hartvig D.L., Møller P. (2011). Comparison of sensory specific satiety and sensory specific desires to eat in children and adults. Appetite.

[18] Duerlund M., Andersen B.V., Byrne D.V. (2021). Sensory Specific Desires Affect and are Affected by Actual Snack Choice. J. Food Nutr. Health.

[19] Meule A. (2020). The Psychology of Food Cravings: The Role of Food Deprivation. Curr. Nutr. Rep..

[20] Griffioen-Roose S., Finlayson G., Mars M., Blundell J.E., de Graaf C. (2010). Measuring food reward and the transfer effect of sensory specific satiety. Appetite.

[21] Rogers P.J., Ferriday D., Irani B., Hoi J.K.H., England C.Y., Bajwa K.K., Gough T. (2020). Sweet satiation: Acute effects of consumption of sweet drinks on appetite for and intake of sweet and non-sweet foods. Appetite.

[22] Mela D.J. (2006). Eating for pleasure or just wanting to eat? Reconsidering sensory hedonic responses as a driver of obesity. Appetite.

[23] Rolls B.J., Hetherington M., Shepard R. (1989). The role of variety in eating and body weight regulation. Handbook of the Psychophysiology of Human Eating.

[24] Duerlund M., Andersen B.V., Grønbeck M.S., Byrne D.V. (2019). Consumer reflections on post-ingestive sensations. A qualitative approach by means of focus group interviews. Appetite.

[25] Duerlund M., Andersen B.V., Byrne D.V. (2019). Dynamic changes in Post-Ingestive Sensations after Consumption of a Breakfast Meal Hign in Protein or Carbohydrate. Foods.

[26] Odermatt A. (2011). The Western-style diet: A major risk factor for impaired kidney function and chronic kidney disease. Am. J. Physiol. Physiol..

[27] Cornil Y., Chandon P. (2016). Pleasure as an ally of healthy eating? Contrasting visceral and Epicurean eating pleasure and their association with portion size preferences and wellbeing. Appetite.

[28] Møller P. (2015). Satisfaction, satiation and food behaviour. Curr. Opin. Food Sci..

